# Public Attention to Mpox in China During the Pandemic: Qualitative Analysis of TikTok Data Using Latent Dirichlet Allocation Topic Modeling

**DOI:** 10.2196/77424

**Published:** 2025-08-21

**Authors:** DongHang Luo, Jie Xu, Yaoyao Jiang, Mengnan Tan, Yaping Yao, Lin He, Jing Ma, Wei Dong, Wei Luo, Chu Zhou

**Affiliations:** 1 National Center for AIDS/STD Control and Prevention, Chinese Center for Disease Control and Prevention, 155 Changbai Road, Beijing, 102206, China, 86 138 1169 6532; 2 Zhejiang Provincial Center for Disease Control and Prevention, Hangzhou, China; 3 Yunnan Provincial Center for Disease Control and Prevention, Kunming, China

**Keywords:** Mpox, TikTok, LDA, Latent Dirichlet Allocation, public perception, health communication, China

## Abstract

**Background:**

Mpox has reemerged as a global public health concern. With the growing reliance on social media for health information dissemination, understanding public perception through these platforms is essential for designing effective health promotion strategies.

**Objective:**

This study analyzes TikTok data related to mpox using Latent Dirichlet Allocation (LDA) topic modeling. This paper aims to extract key topics and inform targeted health promotion strategies for mpox prevention and control.

**Methods:**

Using the “Aisou Jisou” system, we collected TikTok data containing the keyword “Mpox” from April 1, 2022, to March 31, 2025. The dataset comprised 25,672 text data and associated search terms. We analyzed trends in the Search Index and Target Group Index (TGI) across time, gender, age groups, and provinces. LDA topic modeling was applied to identify latent topics within the text data, and topic evolution was examined during 4 peak months of the Search Index.

**Results:**

A total of 4 major Search Index peaks were identified on TikTok in China, which are May 2022, July 2023, August 2024, and February 2025. These peaks aligned with key global and national mpox events, including WHO’s declaration of a global mpox outbreak in May 2022 and the detection of the clade Ib Mpox in China in January 2025. TGI analysis revealed that users aged 18‐23 years exhibited the highest engagement. Spatially, Beijing, Tianjin, and Jilin recorded the highest cumulative TGI values (5922.38, 5692.41, and 3579.90, respectively). LDA topic modeling identified 8 primary topics, including transmission and prevention, vaccine concerns, and misinformation, etc. Public attention evolved from general disease knowledge toward issues of stigmatization and vaccine distrust over time. Sankey diagrams illustrated shifts in public attention across topics at different Search Index peaks, with “Mpox Transmission and Prevention” receiving the most attention in May 2022 and “Mpox Vaccination and Infection Prevention” in February 2025.

**Conclusions:**

TikTok provides real-time insights into public attention during mpox outbreaks, but can also propagate misinformation and stigmatizing narratives. Public health authorities should leverage these platforms for timely communication, actively address misinformation, and mitigate social bias. Tailored strategies are needed to enhance health literacy, minimize stigma, and strengthen outbreak preparedness and response. This study highlights the dual role of social media as both an information source and a potential vector for misinformation, emphasizing the necessity for active monitoring and regulation by health authorities to ensure the accuracy and reliability of disseminated health information.

## Introduction

Mpox, formerly known as monkeypox, is an emerging zoonotic disease caused by the mpox virus (MPXV). The virus was first identified in monkeys in 1958 and has since been detected in various animal species. The first human case was reported in 1970 in the Republic of the Congo in Central Africa. Since then, MPXV has primarily spread within Central and West Africa through animal-to-animal (mainly rodents and nonhuman primates), animal-to-human, and human-to-human transmission pathways [1]. In recent years, globalization has significantly contributed to the cross-border spread of mpox, leading to outbreaks in multiple countries. By May 2022, mpox had spread to 110 countries, prompting the World Health Organization (WHO) to declare it a Public Health Emergency of International Concern (PHEIC) in July 2022. This declaration was lifted in May 2023 as global case numbers declined [2]. However, on August 14, 2024, the WHO again declared a PHEIC due to an outbreak in the Democratic Republic of the Congo, attributed to a previously unreported subtype, clade Ib, associated with more severe disease outcomes [3,4]. As of February 28, 2025, 131 countries have reported a total of 134,034 confirmed cases and 291 deaths globally. Notably, men who have sex with men constituted 86.6% of the cases reported during the preceding 12 months [5].

The first imported case of mpox in mainland China was confirmed on September 16, 2022, in Chongqing [6]. By February 28, 2025, a total of 2849 cases had been documented nationwide, including 549 reported in the preceding year [5]. Although the incidence of mpox in China is lower than that in high-prevalence regions globally, the country’s large population necessitates sustained preparedness for public health challenges associated with potential mpox outbreaks. Previous studies have identified several key risk factors for mpox infection in nonendemic countries, including having multiple sexual partners, close physical contact, MSM status, contact with confirmed cases, and younger age. Vaccination has demonstrated protective efficacy [7]. In China, however, cultural stigma, legal considerations, and the substantial yet often underrepresented MSM population present significant challenges to identifying high-risk individuals [8-10]. In addition, limited public knowledge about mpox has exacerbated fear and impeded the adoption of protective behaviors [11,12]. Therefore, understanding public concerns and formulating targeted health education strategies based on these concerns is crucial for effective outbreak prevention and control [13].

The proliferation of social media platforms represents an inevitable trend in the digital era. As of February 2025, approximately 5.24 billion people—63.9% of the global population—were active on social media [14]. In China, according to the 55th “Statistical Report on China’s Internet Development” released by the China Internet Network Information Center (CNNIC) in January 2025, as of December 2024, the number of Chinese internet users had reached 1.108 billion, with the internet penetration rate rising to 78.6% [15]. As health care communities increasingly shift online, individuals are relying more heavily on the internet for health-related information [16,17]. User interactions on social media platforms, such as comments and shares, reflect public concern and attitudes toward health emergencies [18,19]. TikTok (ByteDance), recognized for its personalized content delivery and algorithm-based recommendations, has gained substantial popularity in China and ranks among the top platforms user activity [20]. According to a report by a third-party research agency QuestMobile, as of December 2024, the combined number of unique active users of TikTok and its lite version reached 978 million, establishing it as a key channel for information dissemination [21]. Despite TikTok’s potential for health information delivery, concerns remain regarding the accuracy and reliability of its content. Some users may disseminate misinformation or incomplete content, potentially triggering unnecessary public panic [22-24].

In this context, this study aims to use Latent Dirichlet Allocation (LDA) topic modeling to analyze mpox-related content on TikTok. LDA topic modeling is an unsupervised Bayesian statistical model widely used in text mining and natural language processing. It represents each document as a mixture of topics, and each topic as a probability distribution over words [25,26]. By applying LDA topic modeling to analyze mpox-related discussions and comments on TikTok, we aimed to gain insights into public information-seeking behaviors and attention toward mpox. The findings provide a scientific basis for developing targeted health communication strategies and interventions. Furthermore, LDA topic modeling can identify knowledge gaps and hotspots in public discourse on TikTok, providing valuable insights not only for mpox prevention but also to the study of public concerns about other diseases. This approach facilitates the optimization of health communication content and strategies, ultimately enhancing public health literacy and preparedness.

## Methods

### Ethical Considerations

All data were sourced from publicly accessible TikTok social media content. Research activities complied strictly with the platform’s terms of service and established ethical guidelines for social media research. No personally identifiable information was collected, and all analyses were conducted on aggregated data to preserve user anonymity.

### Data Sources

The data used in this study were obtained from the “Aisou Jisou” Index Analysis System [27], which provides TikTok-related data (including both TikTok and the “lite version” of TikTok). The system holds a Chinese software copyright registration number from the National Copyright Administration of China, with the registration number being 14092309. The keyword “Mpox” was used for data extraction. The starting point was set to a month before the first reported mpox case in the nonendemic United Kingdom in May 2022, and the endpoint was set to 2 months after the confirmation of the first clade Ib mpox case by the Chinese Center for Disease Control and Prevention in January 2025. The total duration was 3 years, covering the period from April 1, 2022, to March 31, 2025.

Data retrieval involved the following steps: (1) examination of open application programming interfaces, focusing on core endpoints such as “Search Index,” “Associated Search Terms,” Target Group Index (TGI), etc; (2) development of an automated data acquisition program using Java and Python integrated with HTTP/HTTPS protocol, namely “Aisou Jisou” Index Analysis System. This system used browser automation tools (eg, Selenium) and headless browser technology to simulate user interactions for automated monthly API calls; (3) application of reverse engineering and packet analysis techniques for decryption and parsing of potentially encrypted data; and (4) implementation of rigorous data cleaning and preprocessing, including deduplication, missing value handling, data normalization, and outlier detection to ensure data integrity for subsequent analysis.

The retrieved dataset comprised: Search Index, Associated Search Terms, and the TGI stratified by gender, age group, and provinces in mainland China. The Search Index is a proprietary metric developed by TikTok to assess the popularity of specific keywords. It is derived by weighting the search volume of the keyword and its related content. It should be noted that the Search Index does not reflect the exact search volume. The TGI reflects the relative strength or weakness of the consumption behavior of a specific user demographic for a given keyword, as compared to the overall platform user base [28]. A TGI >100 signifies that the proportion of users within the specified demographic engaging with the keyword exceeds the platform average.

### Data Cleaning

Before LDA topic modeling, data underwent preprocessing to ensure quality: removal of stopwords (eg, modal particles or meaningless symbols), standardization of synonyms (eg, uniform expressions for countries or disease abbreviations), phrase merging (eg, combining multiword terms into single tokens), and word segmentation. These procedures minimized noise and enhanced semantic clarity for topic modeling.

### Statistical Analysis

Descriptive analysis of the Search Index was conducted using WPS (Kingsoft Corporation). LDA topic modeling and associated workflows—including data preprocessing, word segmentation, perplexity and coherence evaluation, topic modeling, and visualization—were conducted using Python (version 3.12.0; Python Software Foundation) with the following libraries: “matplotlib,” “numpy,” “jieba,” “scipy,” “gensim,” “pandas,” “pyLDAvis,” “openpyxl,” and “pyecharts.” The “jieba” library is a robust Python tool for Chinese text processing, offering efficient word segmentation, multiple segmentation modes, custom dictionary support, part-of-speech tagging, and keyword extraction capabilities.

Determining the optimal number of topics is critical in LDA topic modeling, as both insufficient and excessive topics can compromise interpretability. Model performance was assessed using perplexity and coherence scores. Lower perplexity values indicate superior predictive performance on unseen data, whereas higher coherence scores correspond to more semantically coherent and interpretable topics [29,30].

For the LDA topic modeling, a corpus of 25,672 text data underwent preprocessing (stopword removal and part-of-speech-based keyword filtering). Subsequently, the LDA topic modeling was applied for topic mining with the following parameters: alpha=0.0125, beta=0.01, iterations=100, chunksize=400, and update_every=1. Model evaluation encompassed perplexity, coherence, and intertopic distance to ensure high topic coherence and interpretability.

**Figure 1.**
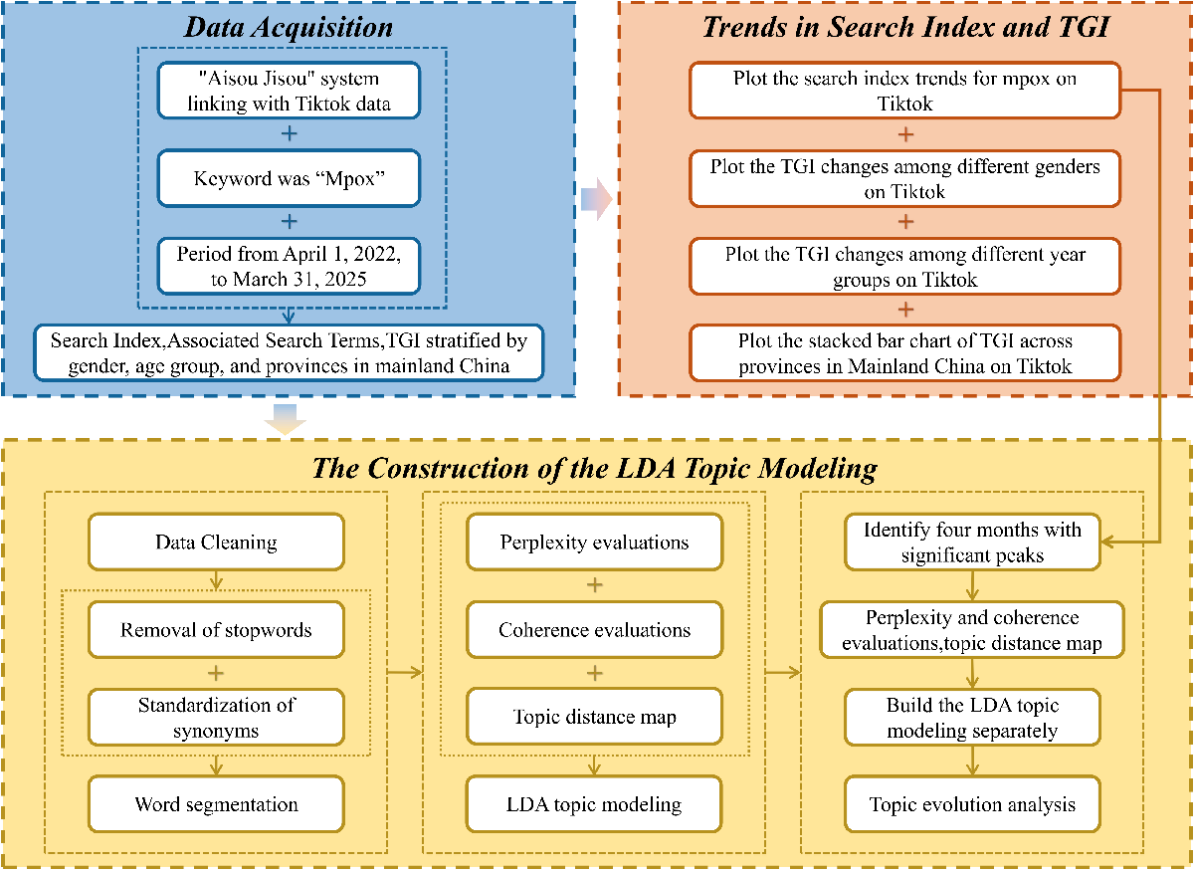
Workflow diagram to explore public attention to mpox in China. TGI: Target Group Index; LDA: Latent Dirichlet Allocation.

Models with topic numbers ranging from 1 to 15 were evaluated. For each model, the top 15 words per topic were identified based on the conditional probability *P(w|z*) (probability of word “w” given topic “z”). Topic term distribution and overlap were visualized using “pyLDAvis.” A nonoverlapping term distribution across topics served as an additional selection criterion. Separate LDA topic modeling was conducted for peak months of public attention (identified via Search Index peaks) to track topic evolution and thematic shifts over time (see Figure 1). Figure 1 illustrates the study methodology.

## Results

### Trends in Search Index and TGI

**Figure 2.**
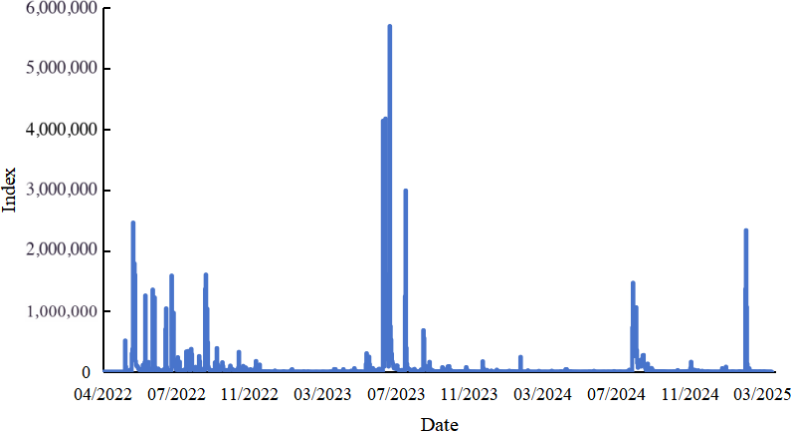
Mpox search index trends on TikTok (April 1, 2022, to March 31, 2025).

From April 1, 2022, to March 31, 2025, the Search Index for “Mpox” on TikTok in China showed 4 significant peaks, corresponding to May 2022, July 2023, August 2024, and February 2025 (see Figure 2). These spikes aligned with major epidemic events both globally and domestically.

**Figure 3.**
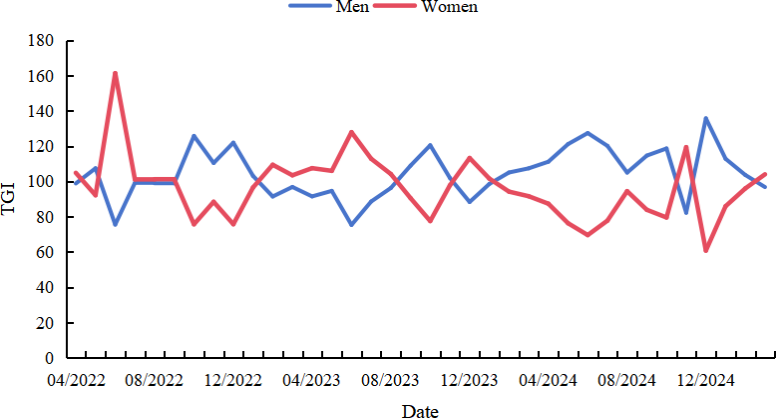
Target Group Index (TGI) changes among different genders on TikTok (April 1, 2022, to March 31, 2025).

TGI analysis revealed fluctuating public engagement across genders without a clear long-term bias; however, male users maintained higher TGI values for extended periods as the outbreak progressed. In terms of age, younger users consistently demonstrated higher TGI, with the 18‐23 years age group exhibiting the highest and most volatile engagement, peaking in late 2024; the 24‐30 years age group followed with moderate but stable attention; while older groups (31‐40 years, 41‐50 years, and 51+ years age group) showed lower and more stable engagement. Spatially, provinces such as Beijing, Tianjin, and Jilin recorded the highest cumulative TGI values of 5922.38, 5692.41, and 3579.90, respectively; individual months also showed sharp increases in regions like Gansu (December 2023) and Tianjin (March 2025), possibly reflecting localized outbreaks or viral content amplification (see Figures3^5^).

**Figure 4.**
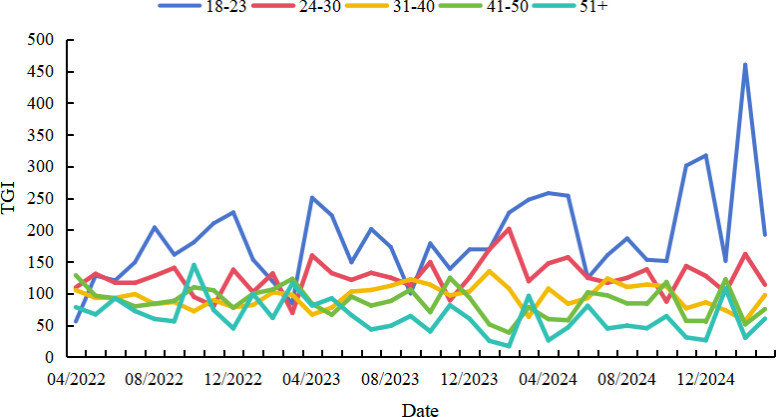
Target Group Index (TGI) changes among different year groups on TikTok (April 1, 2022, to March 31, 2025)

**Figure 5.**
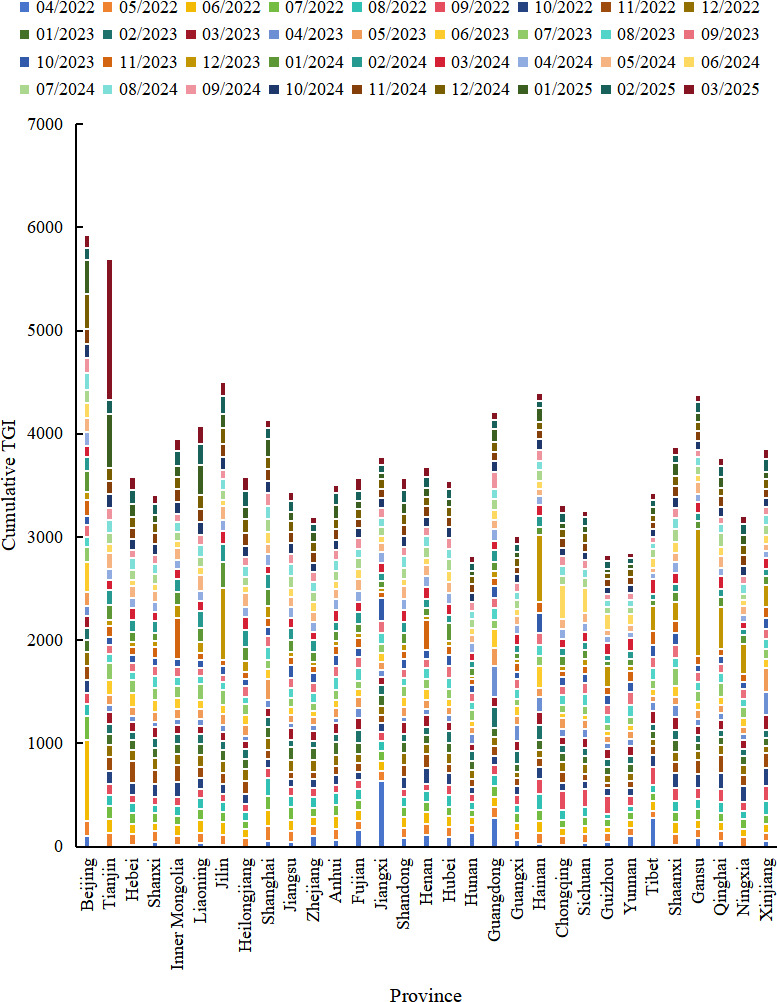
Stacked bar chart of Target Group Index (TGI) across provinces in Mainland China on TikTok (April 1, 2022, to March 31, 2025).

### LDA Topic Modeling Results

**Figure 6.**
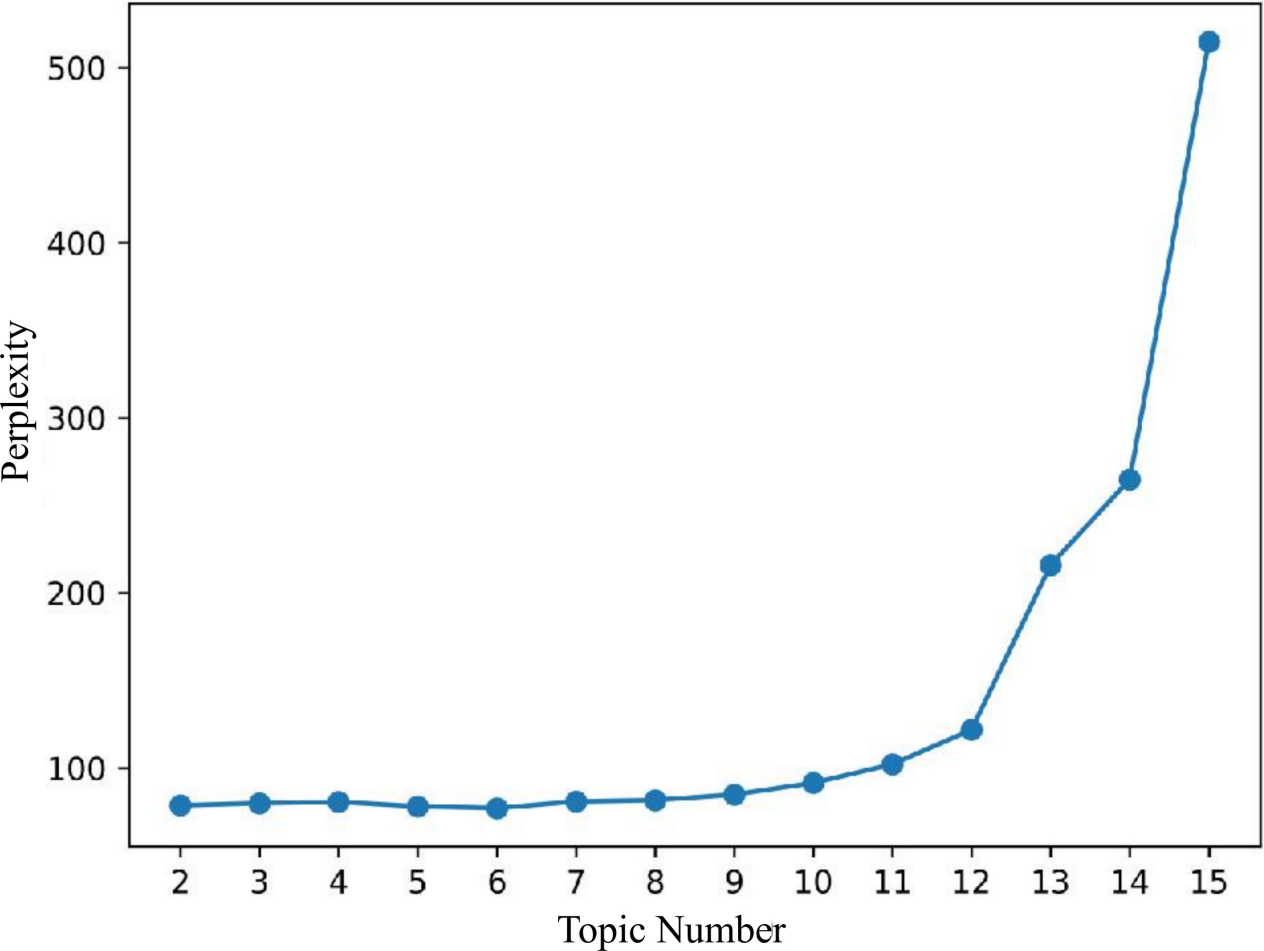
Perplexity scores for different topic counts in Latent Dirichlet Allocation (LDA) topic modeling.

LDA topic modeling, based on perplexity and coherence evaluations (see Figures6  7), determined the optimal number of topics as 8. Topic distance mapping showed minimal overlap (see Figure 8).

**Figure 7.**
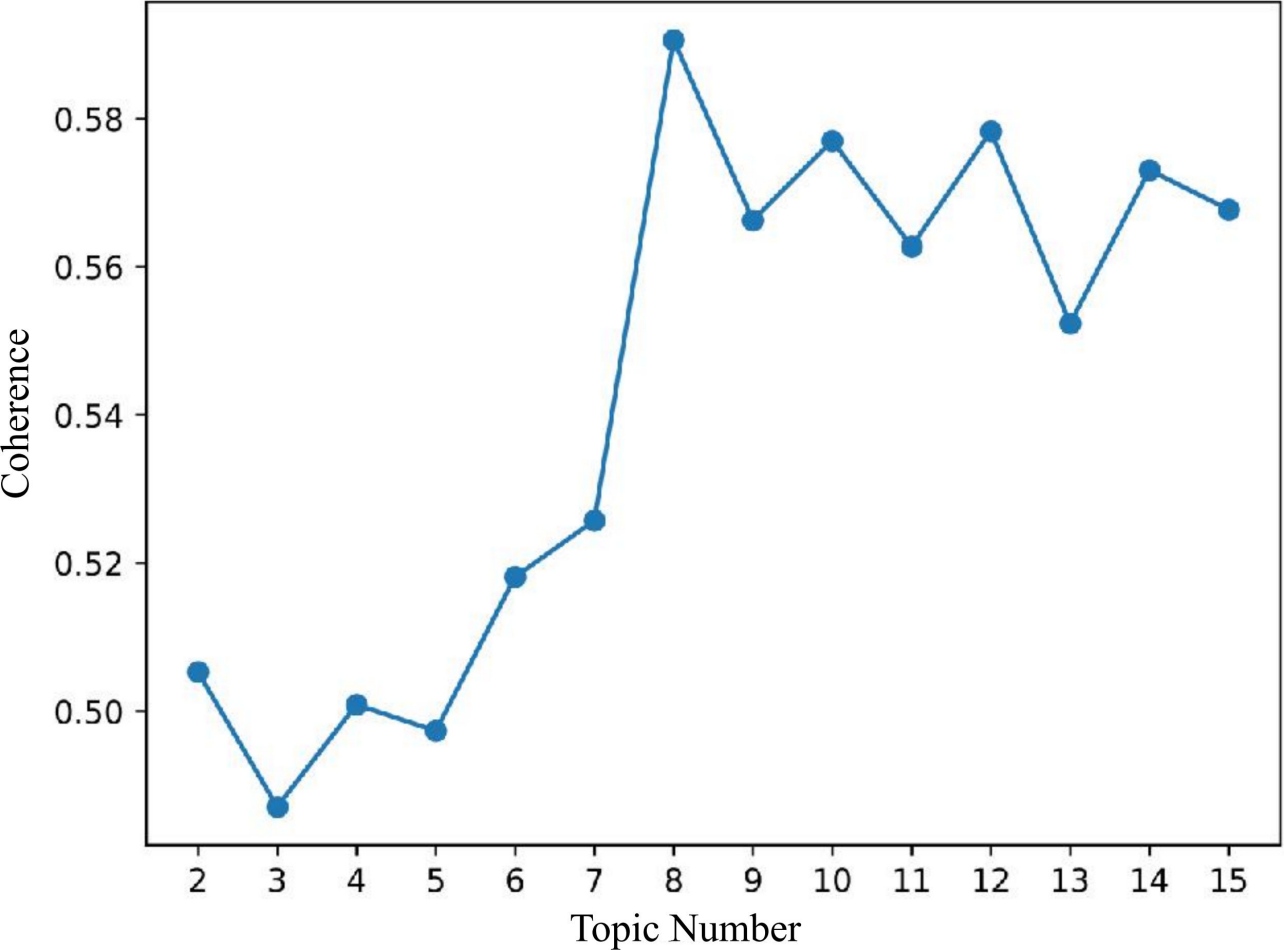
Coherence scores for different topic counts in Latent Dirichlet Allocation (LDA) topic modeling.

**Figure 8.**
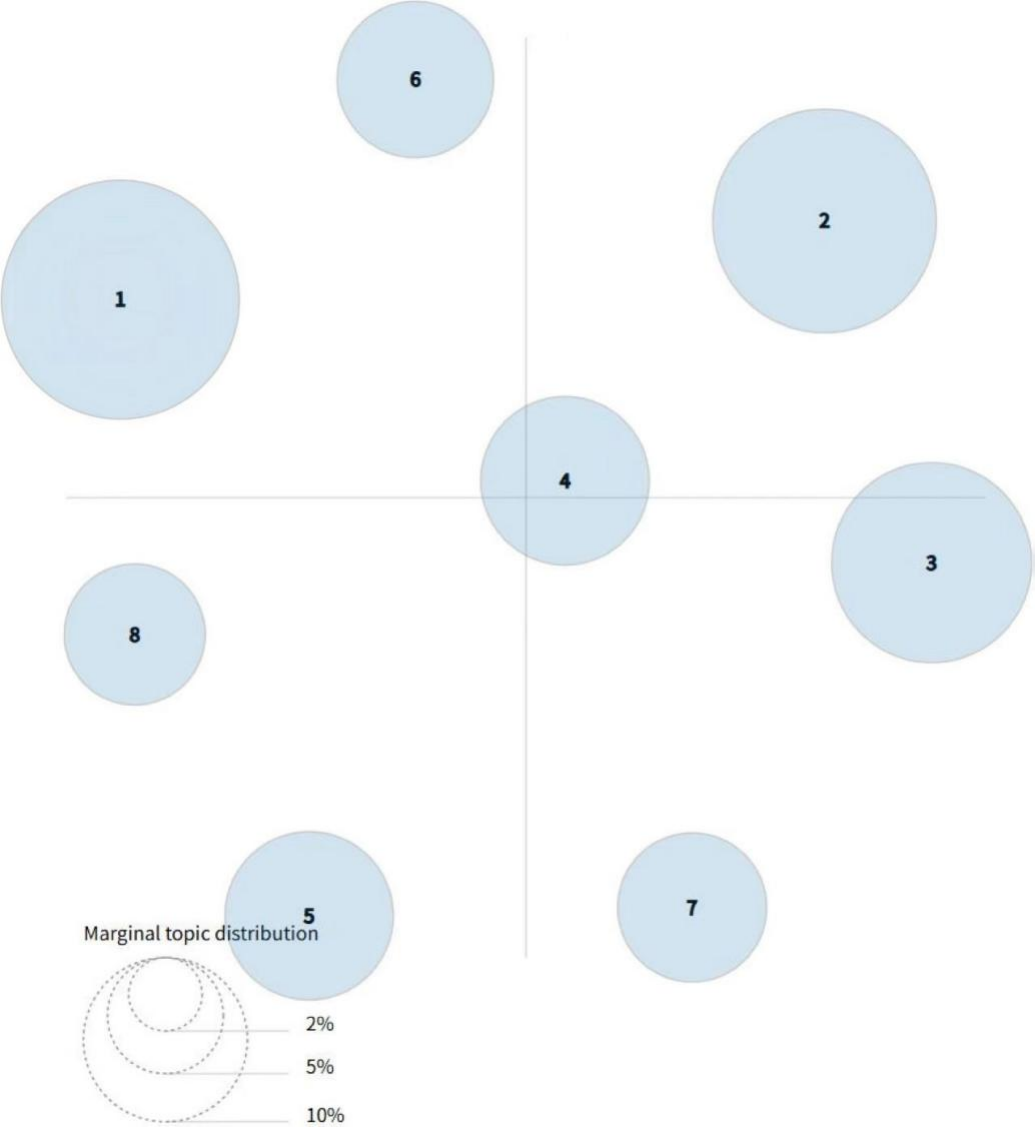
The intertopic distance map for 8 topics in Latent Dirichlet Allocation (LDA) topic modeling.

To present high-frequency and high-probability associated words under each topic, we ultimately selected 15 practically significant associated words for each topic (see Table 1), it presents the top 15 associated words ranked by *P(w|z*) for each topic, along with their term frequencies.

**Table 1.** The word frequency and top 15 associated words of *P*(*w*|*z*) for 8 topics in Latent Dirichlet Allocation (LDA) topic modeling.

Topic number	Topic	Top 15 related words (word frequency, *P*[*w*|*z*]^a^)
1	Mpox transmission and prevention	patients (637, 0.239) rash (259, 0.152) Mpox (606, 0.141) virus-infected persons (178, 0.072) Sichuan (197, 0.067) infected persons (128, 0.067) prevention (310, 0.064) Beijing (171, 0.061) Shenyang (77,<0.05) Shanghai (116,<0.05) rumor-busting (80,<0.05) WHO (78,<0.05) official (63,<0.05) disease (25,<0.05) fever (11,<0.05)
2	Global mpox epidemic monitoring and control measures	Latest news (0.318, 1240) dense (0.115, 469) phobia (0.115, 471) honeycomb (0.092, 274) holes (0.09, 292) detection (0.071, 333) test kit (0.05, 140) CDC (<0.05, 196) growth (<0.05, 77) serious (<0.05, 100) experts (<0.05, 112) interpretation (<0.05, 63) Heilongjiang (<0.05, 44) transmission method (<0.05, 257) condition (<0.05, 31)
3	Disease treatment and prevention strategies	Chickenpox (0.227, 545) difference (0.172, 314) AIDS (0.138, 257) treatment (0.138, 328) recovery (0.056, 186) Changsha (0.051, 128) medicine (0.05, 73) Henan (<0.05, 84) effective (<0.05, 93) distinguish (<0.05, 36) fever (<0.05, 102) protection (<0.05, 133) method (<0.05, 127) effect (<0.05, 16) Mpox (<0.05, 3)
4	Epidemic transmission and prevention measures	Red dot (0.416, 331) outbreak (0.229, 337) droplet (0.062, 102) WHO (0.06, 325) organization (0.057, 262) sexual transmission (<0.05, 35) prevention (<0.05, 47) hands and feet (<0.05, 22) transmission speed (<0.05, 40) warning (<0.05, 23) highest level (<0.05, 24) cooked meat (<0.05, 1) smiling face (<0.05, 1) people (<0.05, 1) life-threatening (<0.05, 1)
5	Epidemic transmission characteristics and control	Infectious (0.167, 614) New (0.133, 481) First case (0.106, 685) Confirmed (0.1, 648) Cases (0.093, 2678) Disfigurement (0.072, 326) Preventive measures (0.064, 177) Mainland (<0.05, 191) AIDS (<0.05, 382) Symptoms (<0.05, 93) Warts (<0.05, 44) Reports (<0.05, 163) Holes (<0.05, 30) Terror (<0.05, 33) Congo (<0.05, 114)
6	Vaccination and infection prevention	infection (0.145, 1300) male (0.111, 446) vaccine (0.095, 845) poxvirus (0.089, 769) COVID (0.088, 507) easy (0.065, 150) USA (0.053, 652) virus (<0.05, 321) smallpox (<0.05, 202) vaccination (<0.05, 96) male (<0.05, 248) hole (<0.05, 139) inclusion (<0.05, 71) female (<0.05, 47) isolation (<0.05, 105)
7	Case detection and transmission research	Cases (0.181, 2678) Discovery (0.169, 1402) Chongqing (0.13, 707) Infectiousness (0.106, 342) Homosexuality (0.063, 126) Importation (0.052, 377) Gay (0.051, 120) Death (<0.05, 292) Foot (<0.05, 86) Strong (<0.05, 84) Variation (<0.05, 311) Not Strong (<0.05, 70) Ratio (<0.05, 90) Infectiousness (<0.05, 614) Disease Energy (<0.05, 73)
8	Symptom presentation and transmission modes	Symptoms (0.297, 1022) Appearance (0.175, 937) Mode of transmission (0.102, 257) Number of people (0.075, 282) Infectious disease (0.074, 263) Class B (<0.05, 123) Statistics (<0.05, 136) STD (<0.05, 72) Infection (<0.05, 1300) Meaning (<0.05, 51) Class A (<0.05, 49) Belong to (<0.05, 69) Cases (<0.05, 2678) Male (<0.05, 16) Specific medicine (<0.05, 89)

a *P (w|z*): conditional probability (probability of word “w” given topic “z”)

We’ve finalized 8 themes based on keyword associations and source text, and labeled them accordingly. For instance, Topic 1 was labeled “Mpox Transmission and Prevention” since its keywords primarily addressed transmission routes and preventive measures. Similarly, Topic 2 was designated “Global Mpox Epidemic Monitoring and Control” as its source text focused on epidemiological surveillance and containment strategies. Corresponding examples for each theme are provided in Table 2.

**Table 2.** Topic labels and examples in Latent Dirichlet Allocation (LDA) topic modeling.

Topic number	Topic	Examples
1	Mpox transmission and prevention	“Mpox outbreak in the Democratic Republic of the Congo” “Will mpox break out in China?” “Will mpox spread widely?”
2	Global mpox epidemic monitoring and control measures	“The latest news on mpox from the China Center for Disease Control” “Mpox detection kit” “How to buy mpox detection kit”
3	Disease treatment and prevention strategies	“Effective treatment for mpox” “How does TCM prevent and treat mpox?” “What does recovery from mpox look like?”
4	Epidemic transmission and prevention measures	“Mpox is transmitted between men and men.” “Is mpox more easily transmitted between homosexuals?” “Isn’t mpox only common among homosexuals?”
5	Epidemic transmission characteristics and control	“Which is more contagious, mpox or COVID-19?” “Is mpox contagious without showing symptoms?” “Is mpox still contagious after recovery?”
6	Vaccination and infection prevention	“Domestic mpox vaccination” “Does China have mpox vaccine” Adding HIV to mpox vaccine”
7	Case detection and transmission research	“First case of new mpox variant found in France” “First case of new mpox variant confirmed in the United States” “Cities in China where mpox cases found”
8	Symptom presentation and transmission modes	“Are the symptoms of mpox and AIDS similar?” “Mpox symptoms pictures” “What are the symptoms of mpox disease?”

Topic labels and interpretations are as follows:

1. “Mpox Transmission and Prevention”: Focused on transmission routes, case distribution, and preventive measures. Keywords such as “patients,” “Mpox,” and “prevention” emphasize geographic spread and control strategies, with frequent references to locations like “Sichuan” and “Beijing.”
2. “Epidemic Monitoring and Control Measures”: Centered on detection methods, diagnostic kits, and public health interventions. Keywords like “detection,” “test kit,” and “CDC” highlight the role of real-time monitoring and institutional response.
3. “Disease Treatment and Prevention Strategies”: Involved topics such as treatment, recovery, and preventive interventions. Terms like “treatment,” “recovery,” and “medicine” suggested a broad discourse on disease management, with comparisons to other diseases like chickenpox and AIDS.
4. “Epidemic Transmission and Prevention Measures”: Emphasized specific routes of transmission (eg, sexual transmission) and corresponding warnings. Keywords suggested a focus on visualized transmission data and international guidance.
5. “Epidemic Transmission Characteristics and Control”: Addressed the dynamic nature of transmission and its severity. Keywords like “infectious,” “first case,” and “preventive measures” emphasized the urgency of outbreak control.
6. “Vaccination and Infection Prevention”: Focused on vaccine efficacy and viral mechanisms. Keywords such as “vaccine,” “infection,” and “poxvirus,” along with references to “COVID” and “smallpox,” highlighted vaccine applications across pathogens.
7. “Case Detection and Transmission Research”: Related to the identification and study of cases and transmission pathways. Keywords like “cases,” “discovery,” and “pathway” reflected attention on early surveillance and viral spread.
8. “Symptom Presentation and Transmission Modes”: Concentrated on clinical symptoms and transmission mechanisms. Keywords like “symptoms,” “mode of transmission,” and “infectious disease” indicated a focus on symptomatology and statistical tracking.

### Topic Evolution During Search Peaks

To explore public interest during peak search months, we conducted separate LDA topic modeling for the 4 significant peak months: May 2022, July 2023, August 2024, and February 2025. Perplexity and coherence metrics were recalculated for each period to determine the optimal number of topics: 2, 6, 4, and 5 topics, respectively (see MultimediaAppendices 1^8^). Topic distances were also examined, and no keyword overlaps were found (see MultimediaAppendices 9^12^).

Multimedia Appendix 13 presents the top 15 keywords for each topic during the 4 peak periods of public interest. In May 2022, users primarily focused on acquiring a general understanding of mpox, encompassing transmission routes, prevention methods, and global outbreak status. By July 2023, public attention had shifted toward real-time news updates, clinical features (eg, incubation period), impacts on specific groups (eg, transgender individuals), and newly reported domestic cases. In August 2024, searches reflected increasing concern regarding visual disease manifestations, medical supply availability, transmission mechanism research, and discussions on vaccine efficacy and administration procedures. By February 2025, attention shifted to China’s latest outbreak status, updated prevention strategies, the initiation of mpox vaccine clinical trials, and extensive media coverage. To visualize thematic transitions across these periods, we constructed Sankey diagrams (see Multimedia Appendix 14) illustrating shifts in user interest in response to evolving epidemic dynamics. Line thickness indicates the strength of thematic linkages between time points. For instance, the topic “Mpox Disease Treatment and Prevention Strategies” (July 2023) evolved from “Mpox Transmission and Prevention” and “Global Mpox Epidemic Monitoring and Control” (May 2022). It subsequently transitioned to “Mpox Outbreak Characteristics and Control in China” and “Mpox Symptom Presentation and Transmission Modes” (August 2024), and further diverged into “Mpox Case Detection and Transmission,” “News Updates on Mpox Prevention,” “Mpox Infection and Treatment in China,” and “Mpox Transmission Characteristics” (February 2025). This analysis demonstrates how mpox-related topics evolved from May 2022 to February 2025, shifting from global surveillance and prevention to regional control and practical applications, with an emphasis on case management and vaccine-based prevention within China.

## Discussion

### The 4 Significant Peaks of the Search Index

In this study, we analyzed the trends in the mpox-related Search Index on TikTok in China between April 1, 2022, and March 31, 2025. A total of 4 significant peaks were identified in May 2022, July 2023, August 2024, and February 2025. These surges corresponded closely with key global and national public health events. Specifically, in May 2022, the WHO announced the beginning of a global mpox outbreak [31]; in July 2023, the Chinese Center for Disease Control reported 106 newly confirmed mpox cases in June, marking the first update since the initial imported case in Chongqing in 2022 [32]; in August 2024, the WHO declared the mpox outbreak in the Democratic Republic of the Congo a PHEIC [3]; and in January 2025, the clade Ib Mpox was detected in China. Subsequently, in February 2025, multiple major Chinese media outlets reposted the latest WHO outbreak report, drawing significant public attention. These findings suggest that public concern regarding mpox on TikTok is highly sensitive to the progression and reporting of the epidemic.

### Younger Users Exhibited Higher TGI

TGI trend analysis by gender and age revealed important demographic insights. Although monthly gender-based TGI values fluctuated, no consistent gender bias was observed. However, male TGI values remained elevated longer as the outbreak evolved, potentially due to the predominance of male patients in China. Notably, age-based differences were more pronounced: younger users had higher TGI, with the 18‐23 years age group showing the most significant engagement and volatility, peaking in late 2024. The 24‐30 years age group followed, with a more stable pattern. In contrast, TGI among users aged 31‐50 years was relatively low with minimal fluctuation, and those aged ≥51 years exhibited the lowest and most stable TGI values. These differences may be attributed to younger users’ higher engagement with social media platforms and their heightened concern regarding emerging health threats. These findings highlight the need for age-targeted health communication strategies. For instance, older adults, who may have limited social media exposure, might benefit more from offline interventions to improve mpox awareness.

### Mpox Epidemic and Influential Individuals May Influence Regional Health Information Dissemination

Geographically, provinces such as Beijing, Tianjin, and Jilin exhibited the highest cumulative TGI values, which could reflect better healthcare infrastructure, more efficient information dissemination, and higher public health literacy. During certain months, individual provinces such as Gansu (December 2023) and Tianjin (March 2025) showed sudden TGI spikes. These may have been triggered by local outbreak reports or intensified media coverage. For example, in December 2023, several widely circulated TikTok videos highlighted Gansu in bold text when discussing new mpox cases [33]. Similarly, in March 2025, a viral post featuring a Weibo screenshot of an mpox case in Tianjin’s Jing’an district gained extensive traction on TikTok [34]. These cases suggest that leveraging influential individuals or visual emphasis in digital communication can enhance the visibility and spread of health-related content.

### Themes and Cognitive Gaps in Mpox-Related Discussions on TikTok

By selecting 8 topics based on perplexity and coherence scores, we were able to comprehensively capture the thematic structure of mpox-related discussions on TikTok. These topics spanned transmission, prevention, surveillance, treatment, vaccination, case reports, and symptomatology—indicating diverse public interests and concerns. However, certain associated keywords reflected ongoing public confusion and challenges to epidemic prevention. The presence of fundamental knowledge gaps was evident. Queries such as “What’s the difference between mpox and chickenpox?,” “Can mpox be cured?,” and “Is mpox still contagious after recovery?” suggest a general lack of understanding. Alarmingly, previous studies have shown that even some sexual health professionals had limited knowledge about mpox [35]. Therefore, enhancing public health literacy and empowering individuals to distinguish accurate from false information are essential to promoting risk awareness and responsible health behaviors.

### Misinformation in Mpox-Related Discussions on TikTok and Responses

Misinformation poses a significant challenge in public health communication during the mpox outbreak. Our analysis identified search terms and topics propagating inaccurate or misleading information. For example, some searches suggested that mpox was spread by U.S. planes or developed by American and Jewish capital (eg, “mpox spread by U.S. planes [36],” “virus developed by American and Jewish capital [37]”). Some search terms and topics indicating public mistrust and concerns about mpox vaccines. For instance, questions like “Why is the mpox vaccine not being promoted?” and “Adding HIV to the mpox vaccine” revealed reluctance and skepticism toward vaccination [10,38]. These false claims distort disease etiology and may incite xenophobic sentiment.

While platforms like TikTok facilitate timely information dissemination, they may also host misleading content. A COVID-19 study reported that 27% of TikTok videos in the early phase contained incorrect or incomplete information [39]. In studies on COVID-19 misinformation [38,40], it has been found that its spread is associated with multiple factors. On the one hand, misinformation can provide the public with an explanation of uncertain events, alleviating their anxiety and thus spreading widely. On the other hand, some groups deliberately tolerate or promote its spread for political, economic, or other vested interests. Thus, content regulation by authorities is essential. Health authorities should therefore actively monitor social media content to curb misinformation. For example, to overcome vaccine hesitancy, it is crucial to enhance public health literacy and provide accurate information about the safety and efficacy of mpox vaccines. In addition, it is imperative for the global political, business, and scientific communities to work together and take coordinated actions to preserve the integrity and credibility of professional expertise and rebuild public trust [40,41].

### Stigma in Mpox-Related Discussions on TikTok and Responses

Stigmatization of affected populations has emerged as a notable issue in the mpox-related discussions on TikTok. Our analysis revealed that some queries and statements perpetuated harmful stereotypes, such as the notion that mpox is only transmitted between men who have sex with men or is common solely among homosexuals. Such stigmatizing discourse can marginalize affected groups, leading to self-concealment of cases and reduced vigilance among the general public. This, in turn, may hinder disease control efforts [9,35]. It is essential to address stigma through education and awareness campaigns that promote understanding and empathy toward affected communities.

### Public Attention Shows Phased Changes at Different Stages of Mpox Epidemic

Public attention also shifted across different stages of the outbreak. In May 2022, when mpox first attracted public attention, searches focused on basic virology and global spread (eg, *“*What kind of disease is mpox?,” “Mpox symptoms look like smallpox,” “Which countries have outbreaks?”). These trends align with previous findings that individuals seek real-time information during health emergencies via social media platforms [42,43].

As public understanding of mpox improved and the outbreak evolved, public attention shifted [44]. In July 2023, keywords such as “expert interpretation of transmission” and “CDC’s mpox infection risk analysis” reflected the growing presence of professionals in content creation. However, stigmatizing discourse also intensified, including inflammatory posts targeting transgender individuals or referencing celebrities associated with LGBTQ+ communities [39,45] (Some comments have linked transgenders to mpox). In August 2024, following the WHO’s renewed declaration of a PHEIC, public discourse once again shifted, with growing expressions of fear—consistent with previous research showing increased fear-related posts during global health emergencies [46,47]. Keywords such as “photos of mpox pustules” and “disfigurement after recovery” exemplify this trend. In February 2025, the release of WHO’s latest mpox report and wide media dissemination reignited public interest, with a focus on domestic developments such as cluster outbreaks, case statistics, and clinical trials of mpox vaccines in China.

Collectively, mpox-related content evolution on TikTok paralleled epidemic progression and public information needs. This supports TikTok’s utility for real-time health surveillance and underscores the need for dynamic health education. Concurrently, public health efforts must counter misinformation, stigmatization, and fear to ensure effective disease control.

### Limitations

Nevertheless, this study has several limitations. First, technical constraints prevented retrieval of all mpox-related TikTok content and more granular analyses of user subgroups. Second, our data were limited to associated search terms, preventing deeper analysis of the content behind those searches. Third, some internal algorithms (eg, TGI calculation) used by TikTok are not fully transparent, which may affect data accuracy. Fourth, TikTok data may not represent nonusers (eg, older adults with limited digital proficiency), introducing coverage bias. Finally, although comprehensive data cleaning was implemented, context-specific or emerging expressions may have been inadvertently excluded by the filtering system.

### Conclusions

This study analyzed TikTok data on mpox in China using LDA topic modeling, revealing that public attention closely followed epidemic developments and varied by age and region. A total of 8 major topics were identified, ranging from transmission and prevention to misinformation and stigmatization. The findings highlight the dual role of social media as both an information source and a potential vector for misinformation. In actual epidemic prevention and control, public health agencies ought to harness TikTok actively. Collaborating with influential bloggers or opinion leaders to devise engaging health information content strategies is a recommended approach, using their sway to steer audiences toward cultivating healthy habits [48]. Meanwhile, establishing a swift monitoring mechanism is essential for detecting misinformation in a timely manner and issuing accurate information to rectify misconceptions [49]. In addition, regularly disseminating authoritative and accurate public health information, maintaining electronic communication with the public to comprehend their needs and concerns, conducting internet-based health education activities, and devising behavioral intervention projects to encourage healthy behavioral changes are also important [50]. Furthermore, analyzing TikTok data to formulate precise communication strategies can elevate the effectiveness of public health communication.

## Supplementary material

10.2196/77424 Multimedia Appendix 1 Perplexity scores for different topic counts in May 2022.

10.2196/77424 Multimedia Appendix 2 Coherence scores for different topic counts in May 2022.

10.2196/77424 Multimedia Appendix 3 Perplexity scores for different topic counts in July 2023.

10.2196/77424 Multimedia Appendix 4 Coherence scores for different topic counts in July 2023.

10.2196/77424 Multimedia Appendix 5 Perplexity scores for different topic counts in August 2024.

10.2196/77424 Multimedia Appendix 6 Coherence scores for different topic counts in August 2024.

10.2196/77424 Multimedia Appendix 7 Perplexity scores for different topic counts in February 2025.

10.2196/77424 Multimedia Appendix 8 Coherence scores for different topic counts in February 2025.

10.2196/77424 Multimedia Appendix 9 The intertopic distance map in July 2023.

10.2196/77424 Multimedia Appendix 10 The intertopic distance map in May 2022.

10.2196/77424 Multimedia Appendix 11 The intertopic distance map in August 2024.

10.2196/77424 Multimedia Appendix 12 The intertopic distance map in February 2025.

10.2196/77424 Multimedia Appendix 13 The top 15 associated words of P(w|z) across different search index peak periods in Latent Dirichlet Allocation (LDA) topic modeling.

10.2196/77424 Multimedia Appendix 14 A Sankey diagram showing the evolution of mpox-related search keyword topics on TikTok across different search index peak periods. (Note: The Sankey diagram illustrates the evolution of TikTok search-query themes related to mpox from May 2022 to February 2025. The text labels summarize each theme’s core content. Ribbons connecting themes across consecutive time points indicate the persistence or shift of attention.)

## Abbreviations

CNNIC: China Internet Network Information Center
LDA: Latent Dirichlet Allocation
MPXV: mpox virus
PHEIC: Public Health Emergency of International Concern
PHEIC: Public Health Emergency of International Concern
TGI: Target Group Index
WHO: World Health Organization
