# Supplementary figures and images for: Public Attention to Mpox in China During the Pandemic: Qualitative Analysis of TikTok Data Using Latent Dirichlet Allocation Topic Modeling
Source: J Med Internet Res. 2025 Aug 21;27:e77424. doi: 10.2196/77424 (PMC12369990; doi:10.2196/77424)

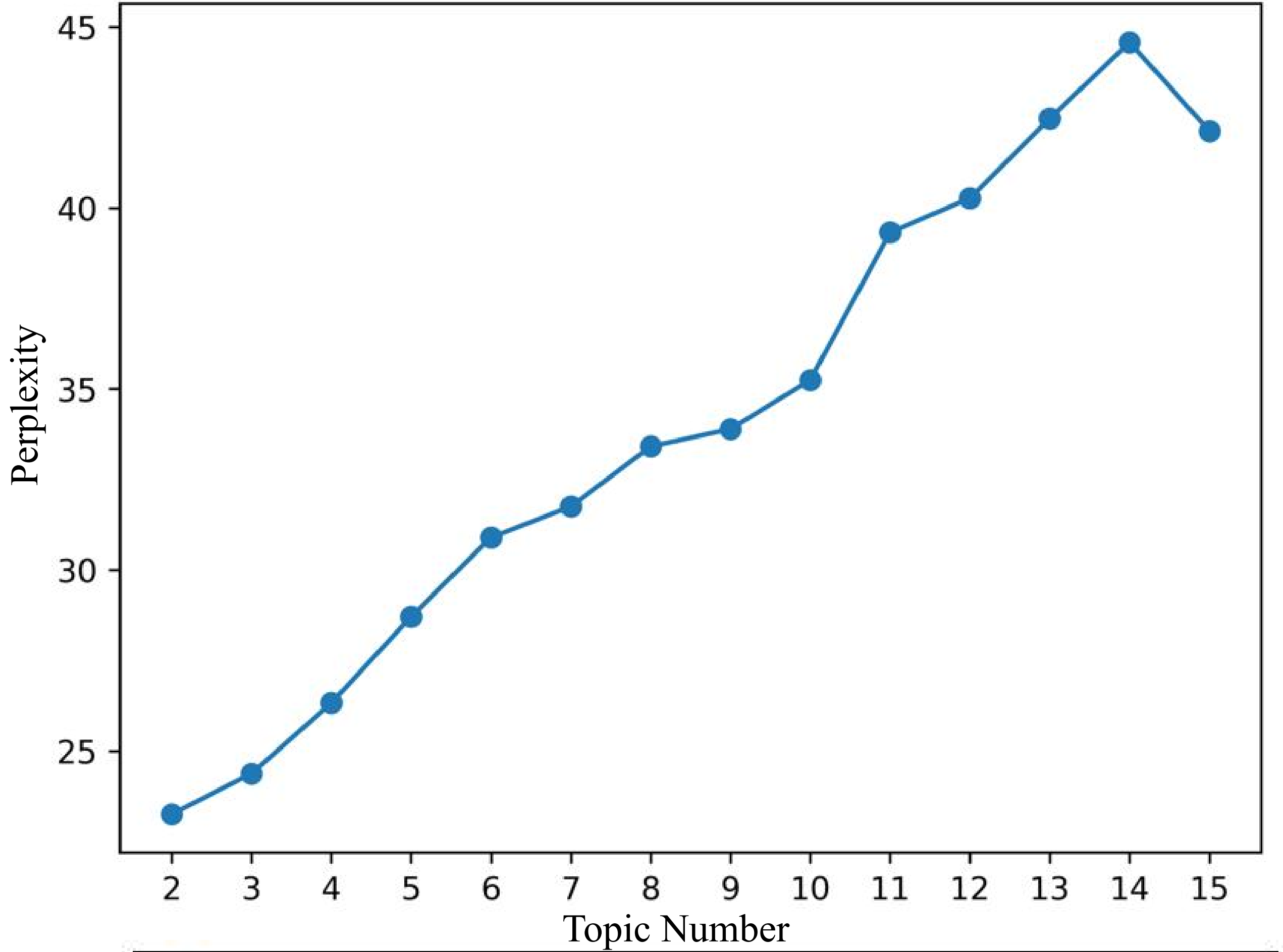

Supplement: Multimedia Appendix 1 [file jmir-v27-e77424-s001.png]

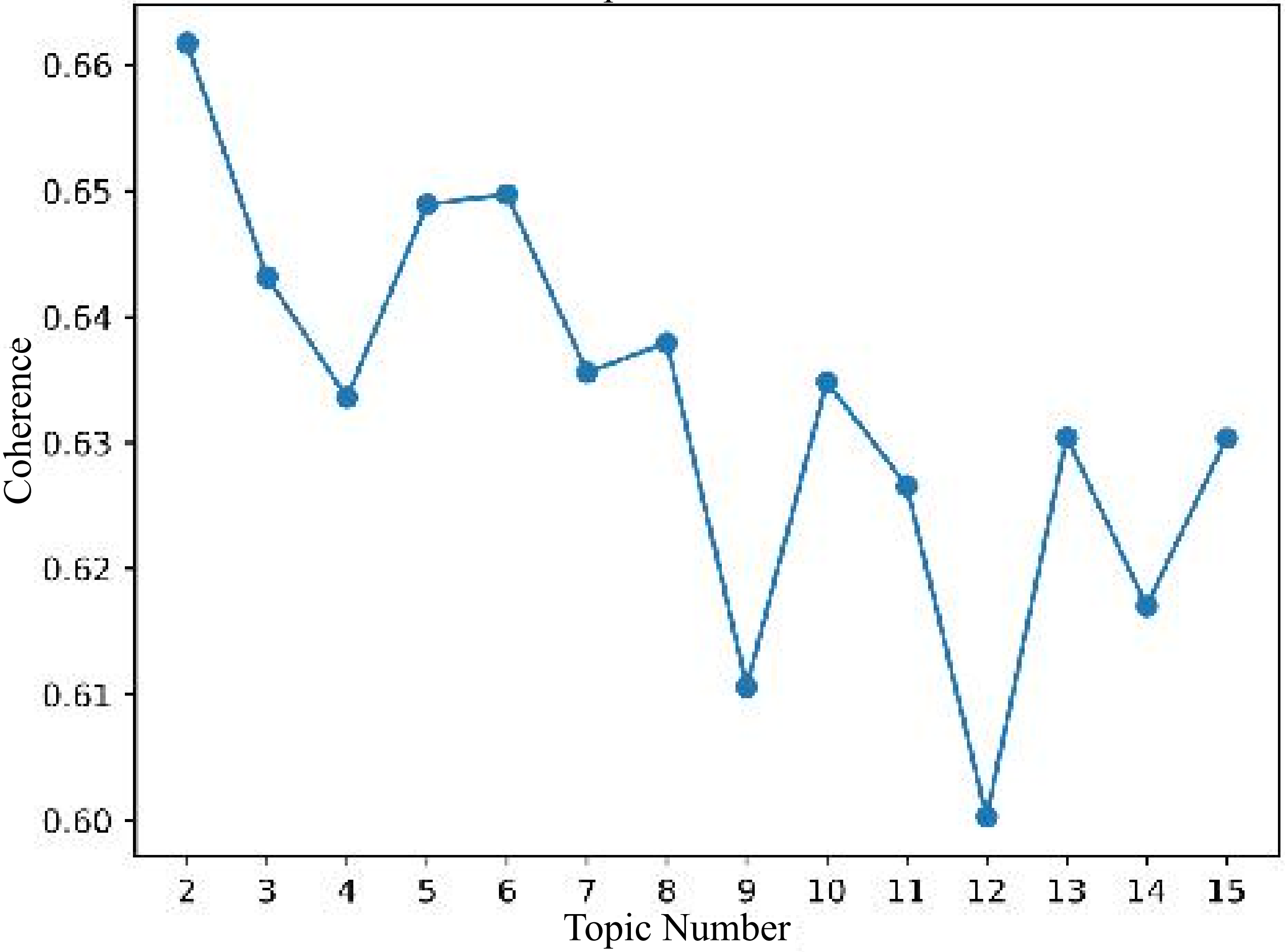

Supplement: Multimedia Appendix 2 [file jmir-v27-e77424-s002.png]

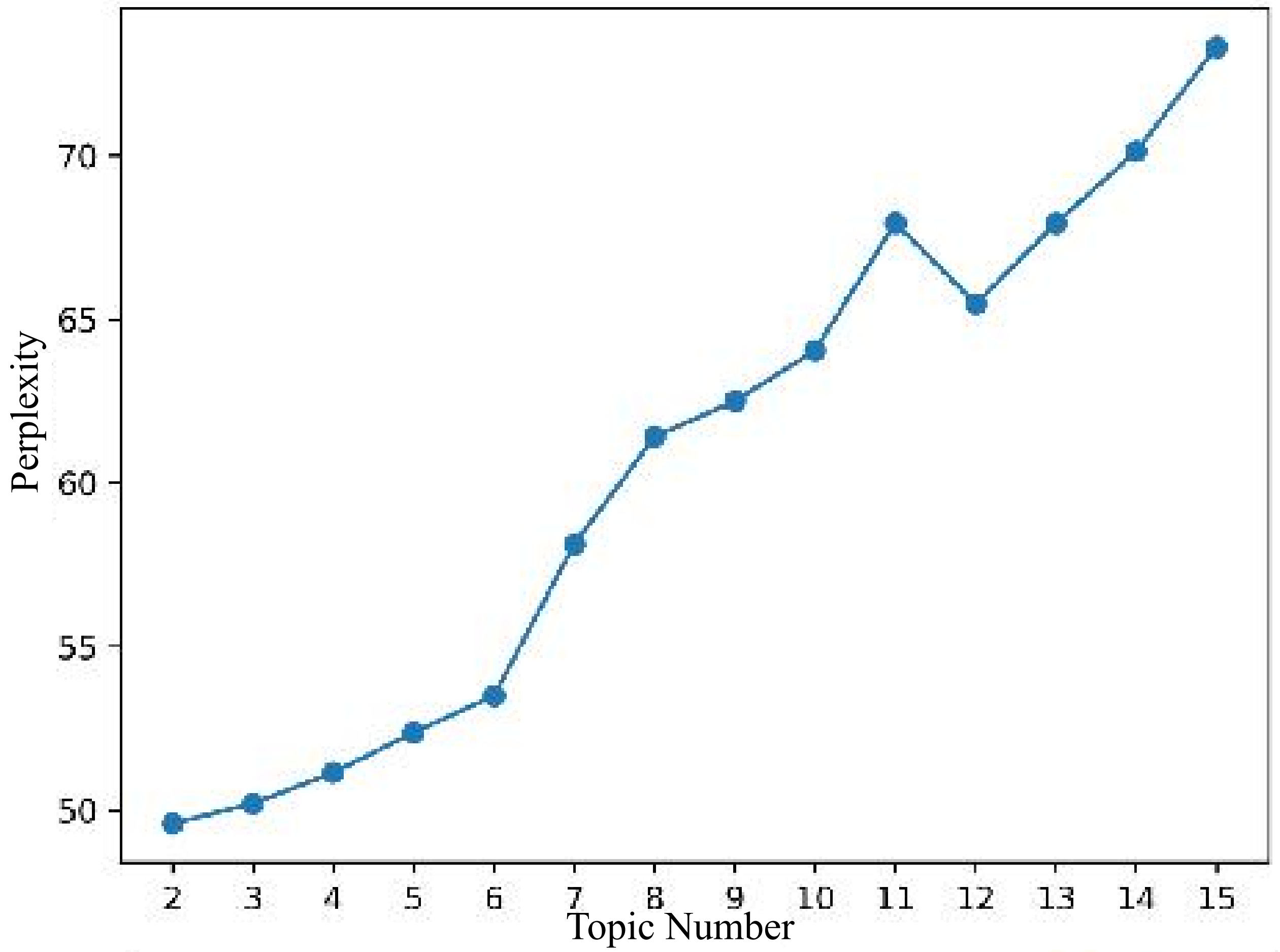

Supplement: Multimedia Appendix 3 [file jmir-v27-e77424-s003.png]

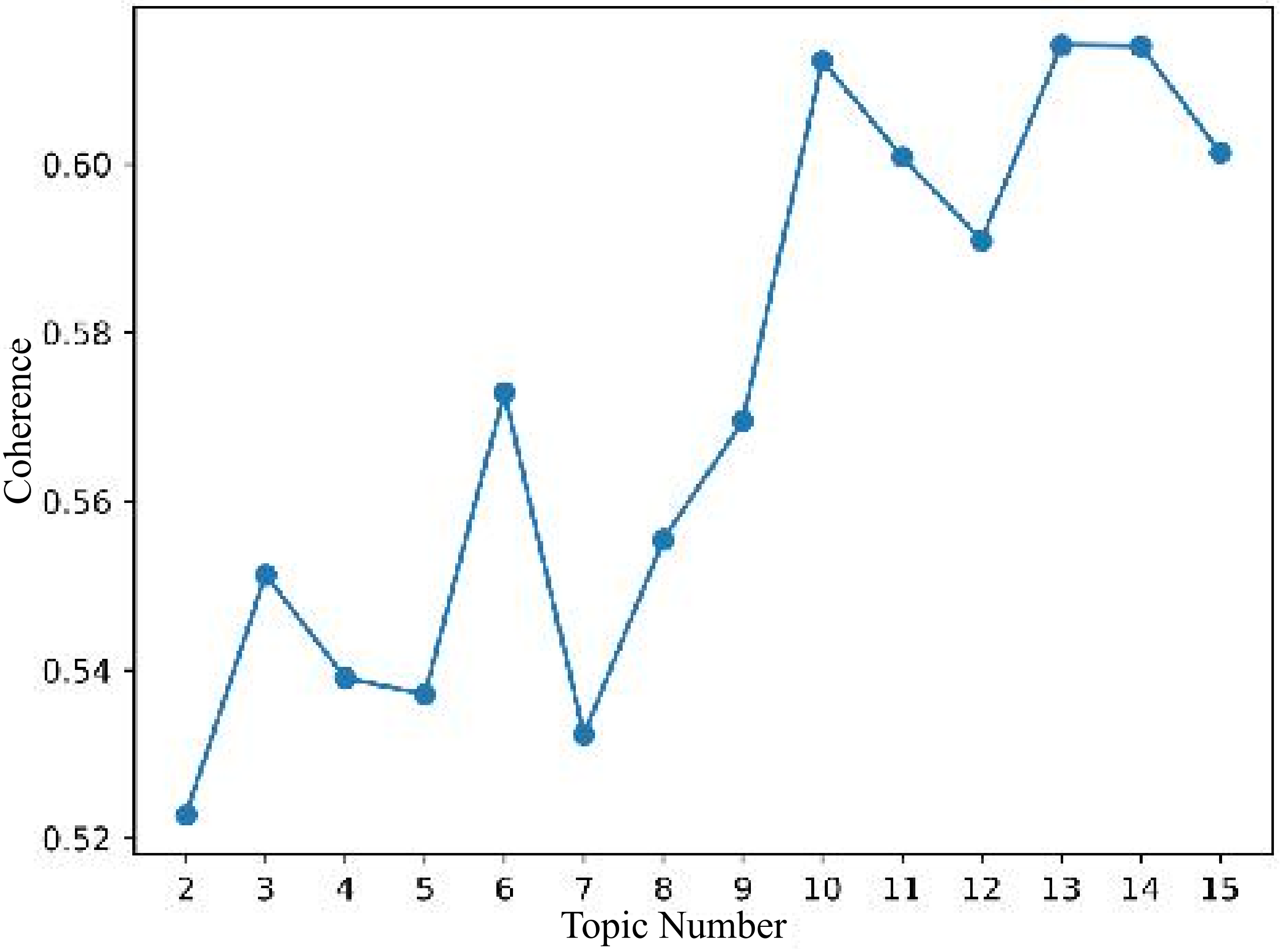

Supplement: Multimedia Appendix 4 [file jmir-v27-e77424-s004.png]

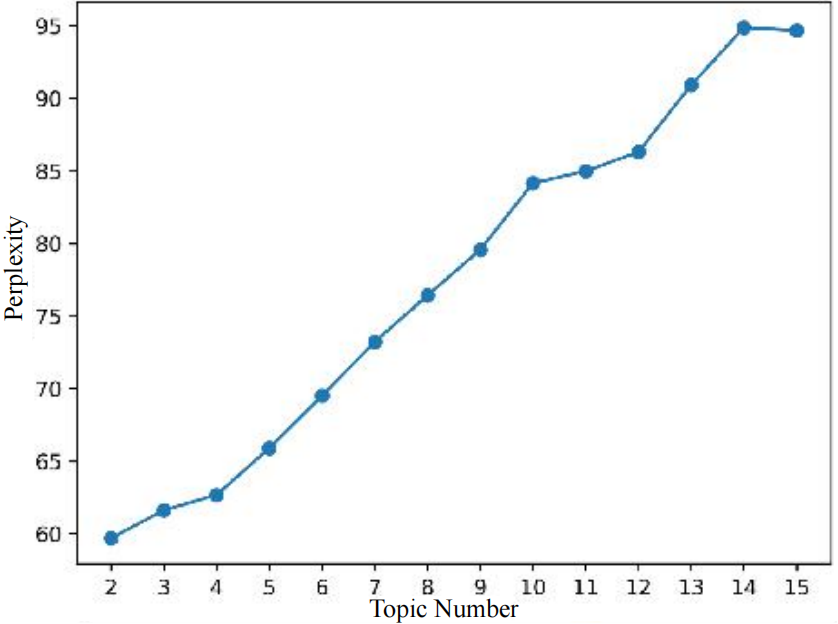

Supplement: Multimedia Appendix 5 [file jmir-v27-e77424-s005.png]

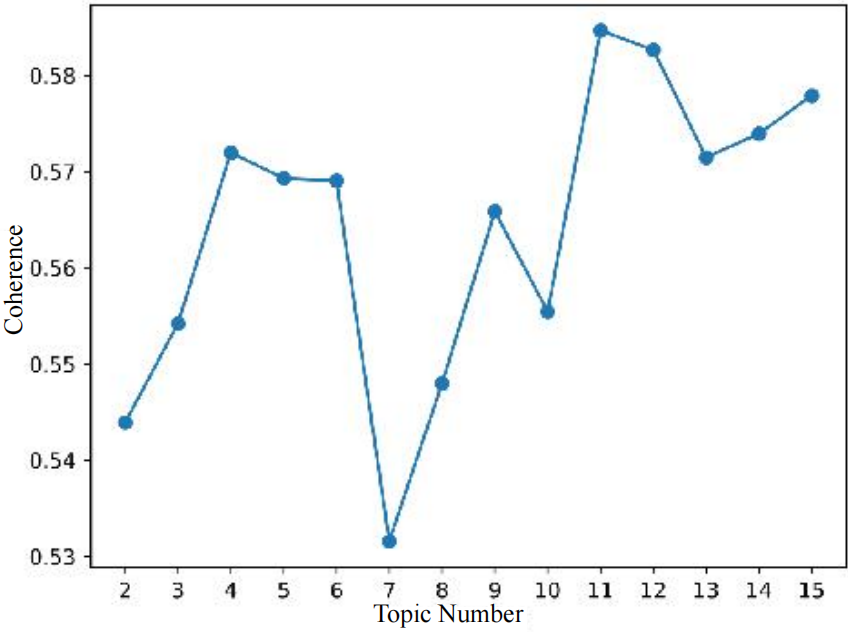

Supplement: Multimedia Appendix 6 [file jmir-v27-e77424-s006.png]

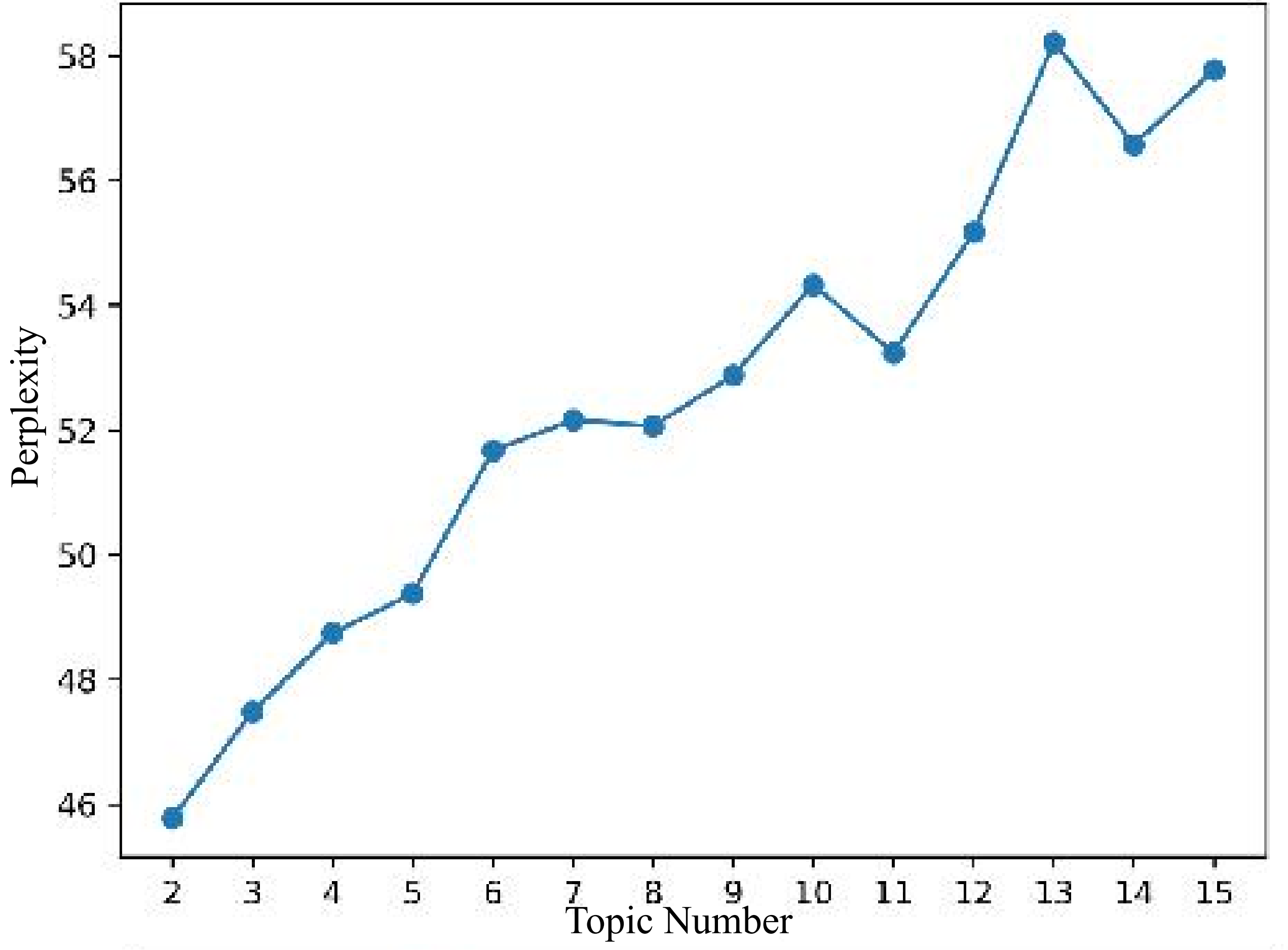

Supplement: Multimedia Appendix 7 [file jmir-v27-e77424-s007.png]

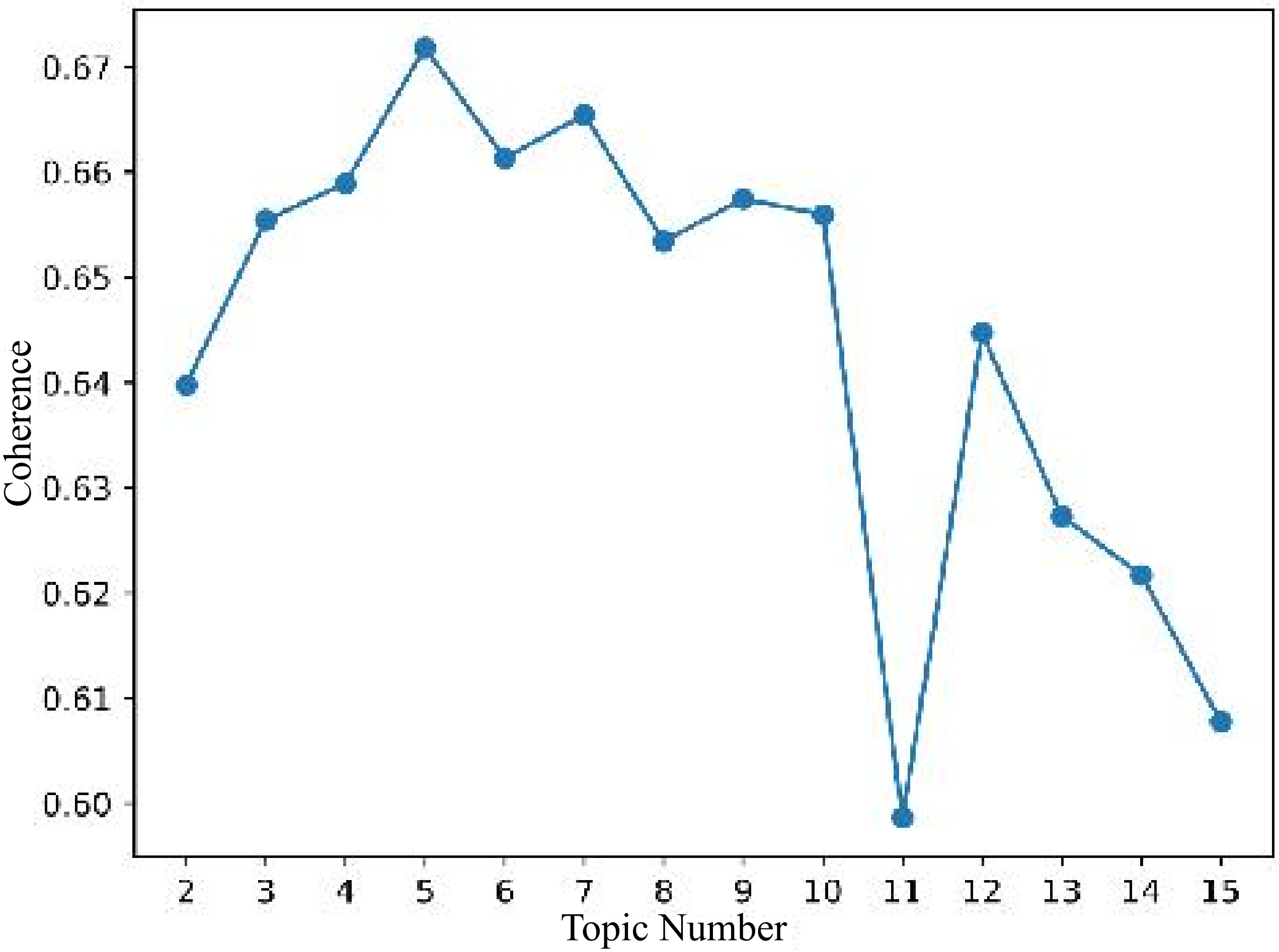

Supplement: Multimedia Appendix 8 [file jmir-v27-e77424-s008.png]

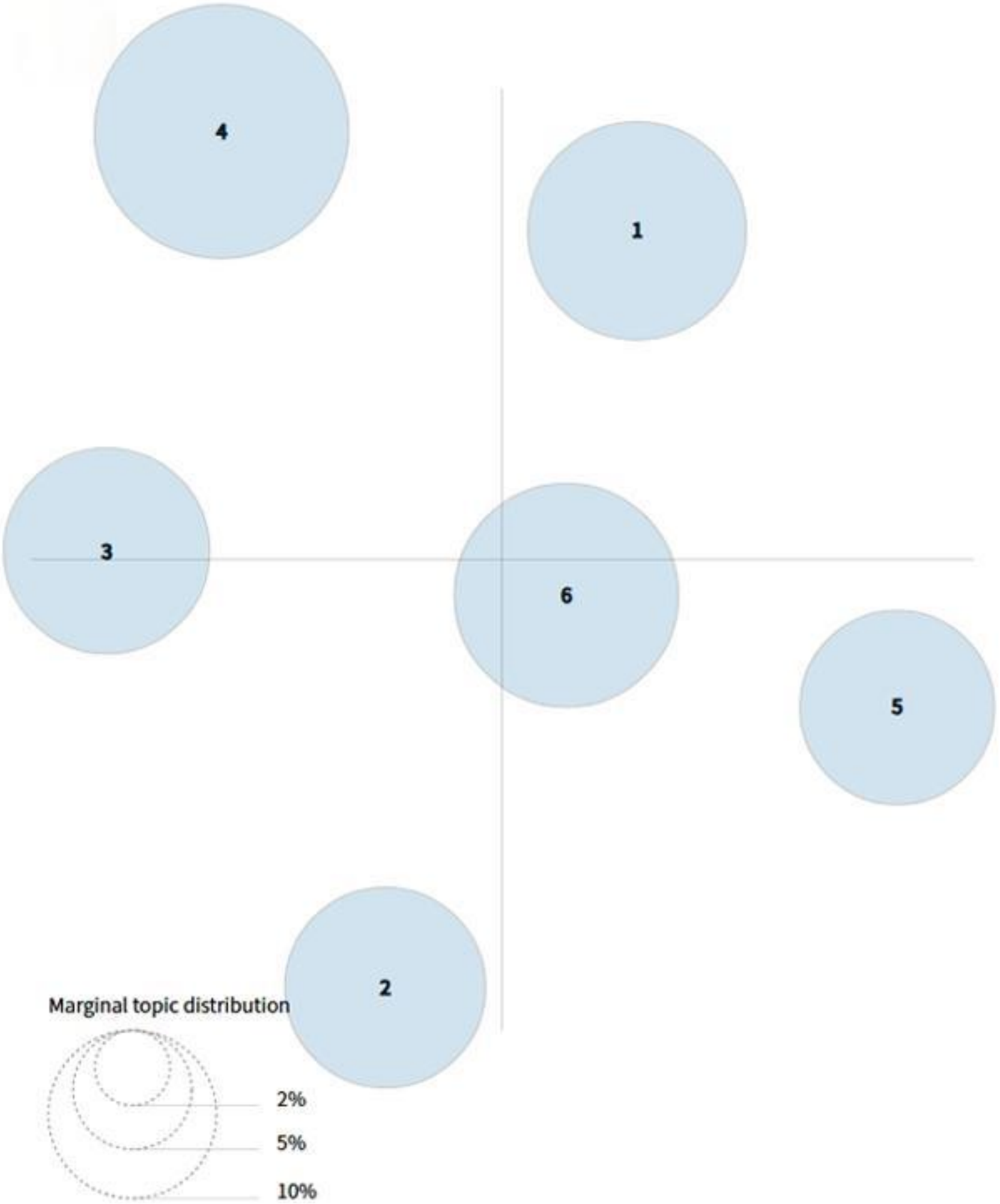

Supplement: Multimedia Appendix 9 [file jmir-v27-e77424-s009.png]

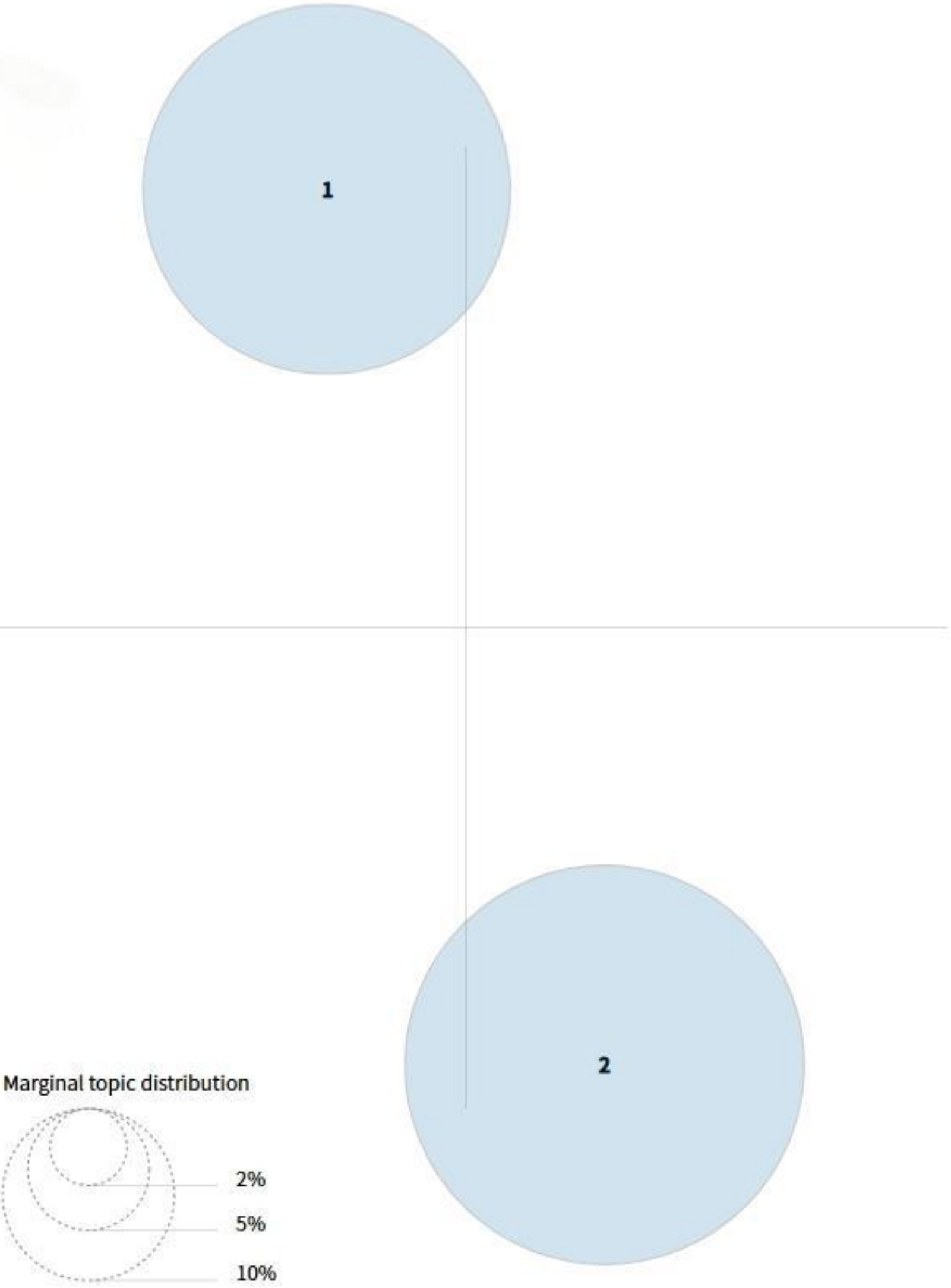

Supplement: Multimedia Appendix 10 [file jmir-v27-e77424-s010.png]

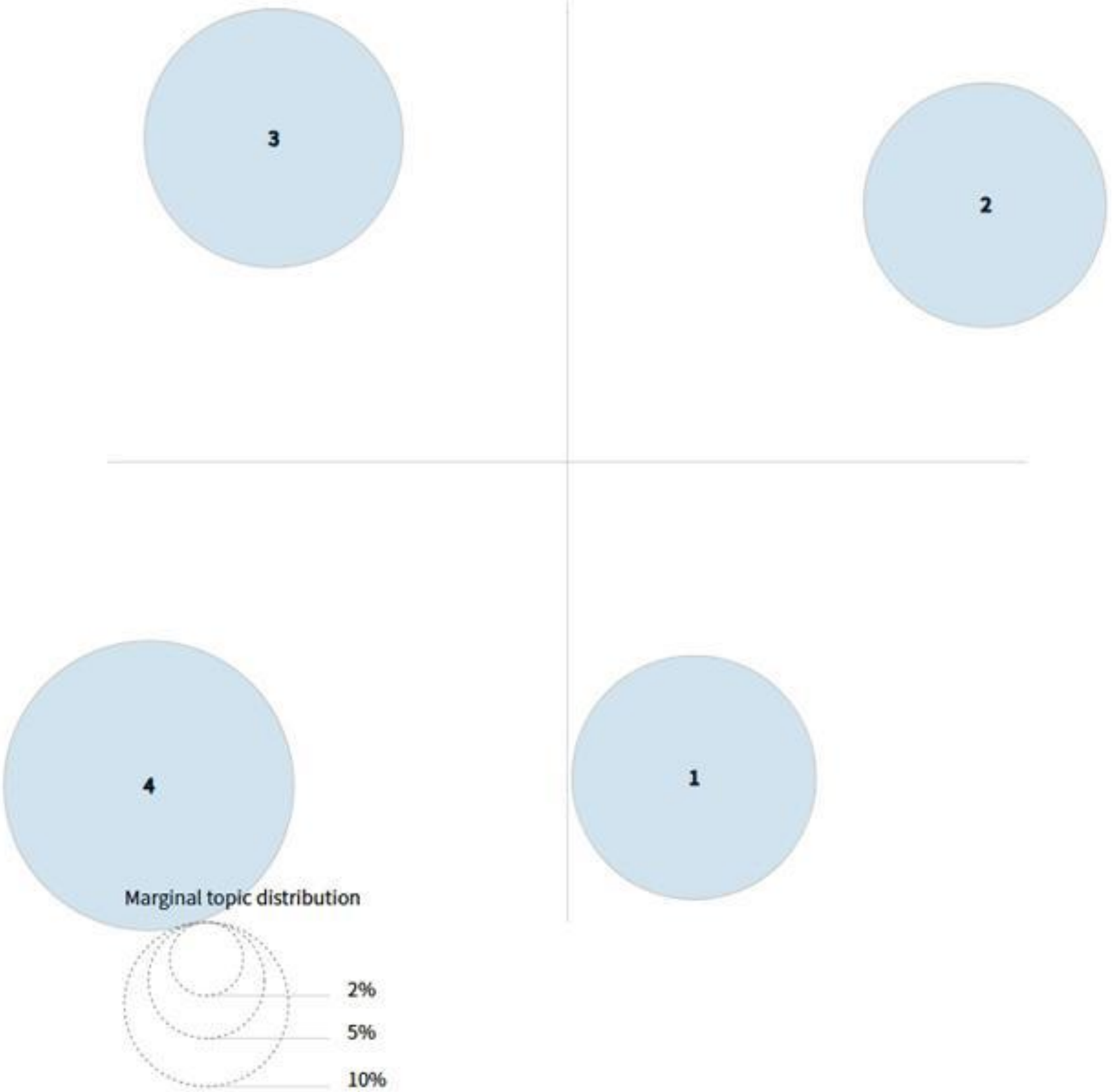

Supplement: Multimedia Appendix 11 [file jmir-v27-e77424-s011.png]

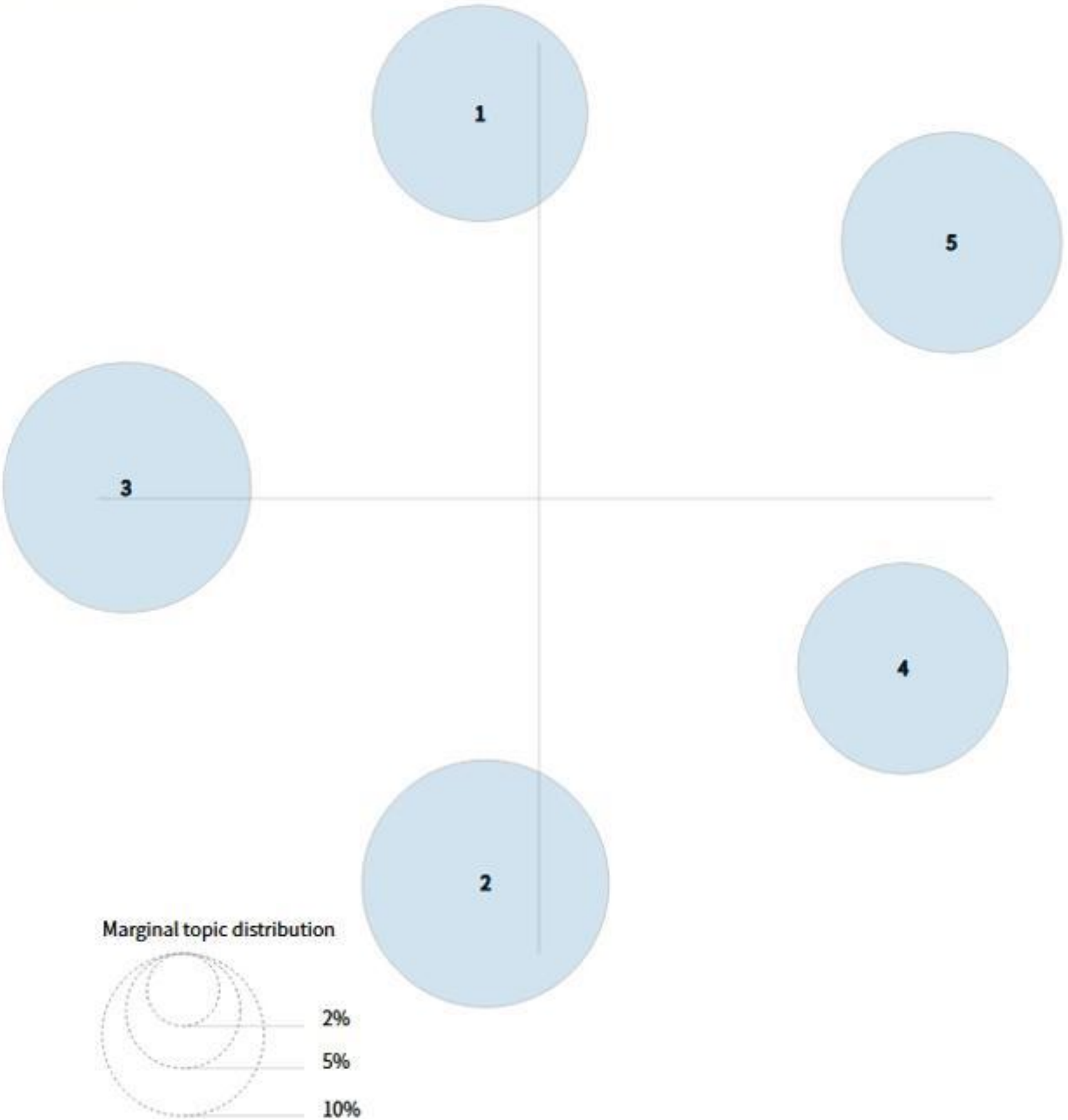

Supplement: Multimedia Appendix 12 [file jmir-v27-e77424-s012.png]

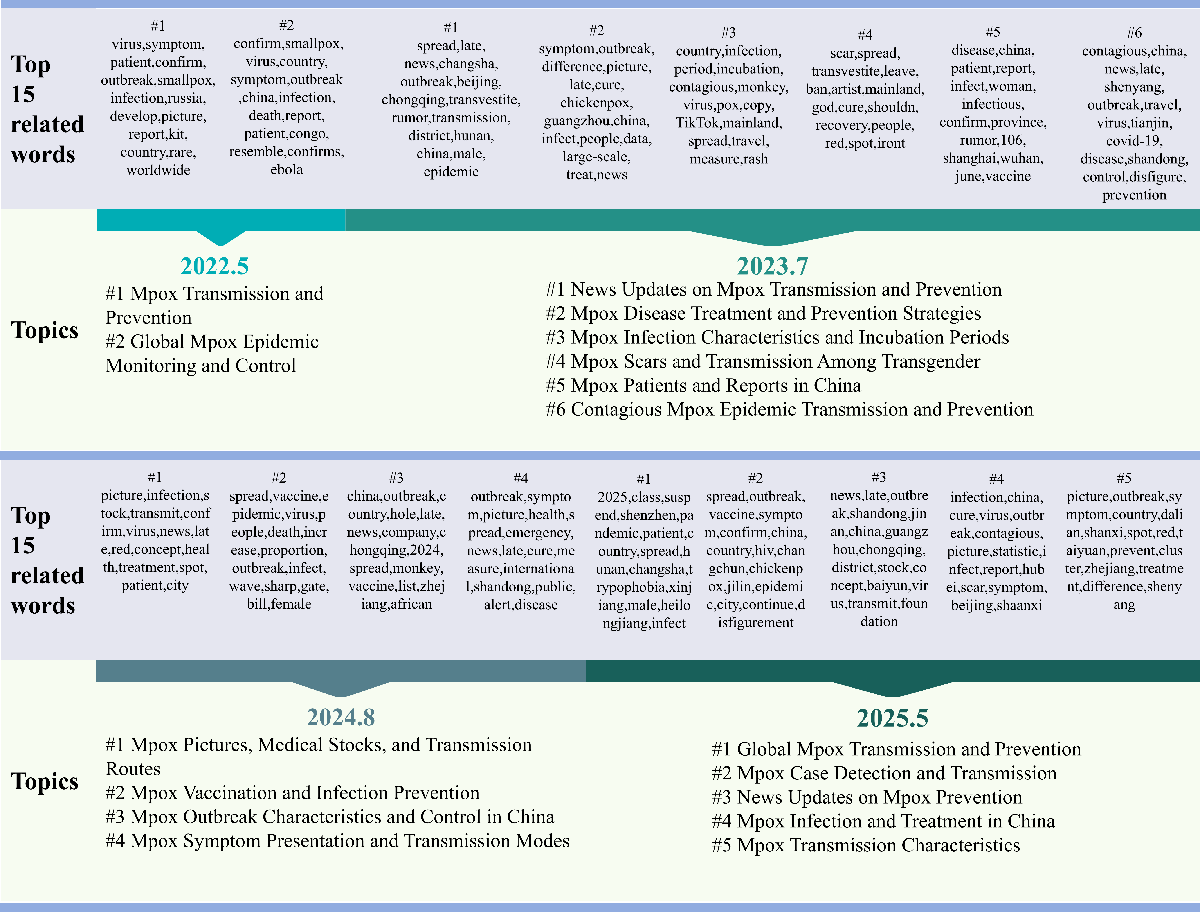

Supplement: Multimedia Appendix 13 [file jmir-v27-e77424-s013.png]

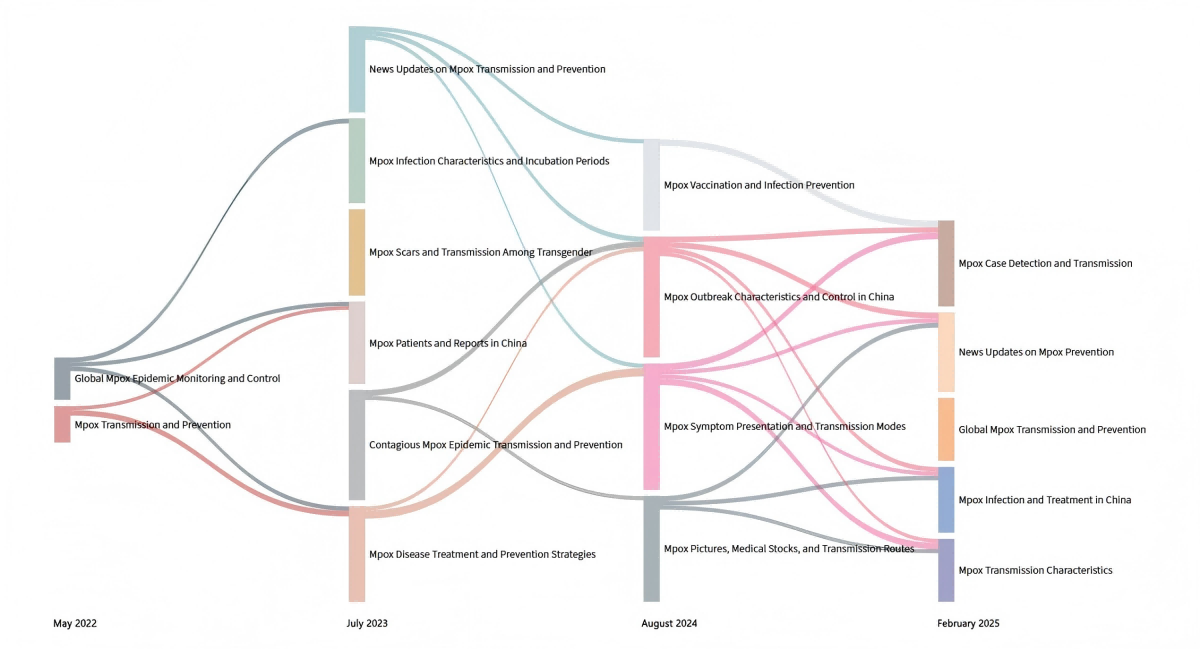

Supplement: Multimedia Appendix 14 [file jmir-v27-e77424-s014.png]
